# A review of spline function procedures in R

**DOI:** 10.1186/s12874-019-0666-3

**Published:** 2019-03-06

**Authors:** Aris Perperoglou, Willi Sauerbrei, Michal Abrahamowicz, Matthias Schmid

**Affiliations:** 1 Department of Mathematical Sciences, University of Essex, Colchester, UK; 2 Institute of Medical Biometry and Statistics, Faculty of Medicine and Medical Center, University of Freiburg, Freiburg, Germany; 3 McGill University Health Centre, McGill University, Montreal, Canada; 4 Medical Biometry, Informatics and Epidemiology, Faculty of Medicine, University of Bonn, Bonn, Germany

**Keywords:** Multivariable modelling, Functional form of continuous covariates

## Abstract

**Background:**

With progress on both the theoretical and the computational fronts the use of spline modelling has become an established tool in statistical regression analysis. An important issue in spline modelling is the availability of user friendly, well documented software packages. Following the idea of the STRengthening Analytical Thinking for Observational Studies initiative to provide users with guidance documents on the application of statistical methods in observational research, the aim of this article is to provide an overview of the most widely used spline-based techniques and their implementation in R.

**Methods:**

In this work, we focus on the R Language for Statistical Computing which has become a hugely popular statistics software. We identified a set of packages that include functions for spline modelling within a regression framework. Using simulated and real data we provide an introduction to spline modelling and an overview of the most popular spline functions.

**Results:**

We present a series of simple scenarios of univariate data, where different basis functions are used to identify the correct functional form of an independent variable. Even in simple data, using routines from different packages would lead to different results.

**Conclusions:**

This work illustrate challenges that an analyst faces when working with data. Most differences can be attributed to the choice of hyper-parameters rather than the basis used. In fact an experienced user will know how to obtain a reasonable outcome, regardless of the type of spline used. However, many analysts do not have sufficient knowledge to use these powerful tools adequately and will need more guidance.

**Electronic supplementary material:**

The online version of this article (10.1186/s12874-019-0666-3) contains supplementary material, which is available to authorized users.

## Background

### Role of splines in modern biostatistics

With progress on both the theoretical and the computational fronts the use of spline modelling has become an established tool in statistical regression analysis. In particular, splines are regularly used for building explanatory models in clinical research. Indeed, many new methodological developments in modern biostatistics make use of splines to model smooth functions of interest, including e.g. non-linear effects of continuous covariates, avoiding distributional assumptions and modelling time-dependent effects in survival analysis, time series, cumulative effects and frequency distributions. For example, searching for the term “splines” at the websites of the journals Statistics in Medicine, Statistical Methods in Medical Research and Biometrical Journal yielded 861, 223 and 189 results, respectively, as of November 24, 2018. Similarly, searching for “splines” in the journals Journal of Clinical Oncology and New England Journal of Medicine (just to name a few) resulted in 156 and 63 hits, respectively, showing that spline modelling is not only important in statistical methods development but is also widely used in applied clinical research. At nature.com, searching for “splines” yielded 2945 results.

An important prerequisite for spline modelling is the availability of user friendly, well documented software packages. In this work, we focus on the R Language for Statistical Computing [33], which has become a hugely popular statistics software since the late 1990’s and which implements a large number of spline functions and modelling options. The implementation of spline and GAM fitting routines has a long tradition in R, since some of the earliest routines were written in the S language, which forms the basis of R [2]. R is not only becoming increasingly popular in applied medical research but is also widely used in university teaching. Moreover, several online resources, blogs and newspapers, report on the popularity of R for data analysis and list it as one of the top programming languages [5, 16, 21, 22, 30, 32, 36]. What makes R so popular is that users can improve and add to the code by writing their own packages, which is then freely available to other users. However, open source software comes with some risks, since it relies on users identifying errors or bugs within packages. This induces a risk that some R packages and routines may not be sufficiently validated and some may fail to provide correct results for specific data structures. An additional challenge for users comes from the fact that help files are also created by individual authors, and do not necessarily meet a standard set of criteria. Although CRAN requires the basic documentation of all functions to be contained in the submitted packages, help files are often not detailed enough to fully understand how the implemented methods work.

In view of these considerations, and following the idea of the STRATOS initiative [25] to provide users with guidance documents on the application of statistical methods in observational research, the aim of this article is to provide an overview of the most widely used spline-based techniques and their implementation in R. Following an introduction to spline modelling and an overview of the most popular spline functions, we will identify and illustrate the use of a set of the relevant R packages. Special focus will be given to the selection and optimization of tuning parameters. Throughout, the paper we will describe methods in a mostly non-mathematical fashion, keeping notation as simple as possible. For mathematical and technical details, we refer to [11, 13, 37, 38, 41].

### About this project

The number of R packages available to users increases exponentially [22]. When version 2.11 of the R software was released in May 2010, there existed 2445 packages available on CRAN. In May 2015, when topic group 2 (TG2) of the STRATOS began the current investigation, CRAN had a little more than 6200 packages available. A simple program was created to search all help files and identify which of these packages contained the word “spline” in the help file. A total of 519 packages were found, out of which 229 packages were identified as relevant to the purposes of this study. All of these packages may (or may not) be interconnected, in the sense that a regression package might require a spline basis package to be loaded before. Packages which are ‘isolated’ usually contain functions to complement a specific research paper, or functions that correspond to a rather specialized method relevant only to a small number of researchers. By May 2016, there were 8670 packages available on CRAN. The increase in the number of new packages illustrates how difficult it is to keep up to date with statistical software, test and evaluate the code and come up with reasonable recommendations. In November 2018 the same number has risen to 13,382 packages contributed by 7642 authors.

In this work, only packages that have a target audience of applied researchers working with regression models will be considered. An important aspect of this project is to identify which are the commonly used methods and inspect what are the outputs of the code when it is applied using default values. The paper targets applied researchers that may have difficulties understanding and calibrating a spline fitting approach. One of the goals here will be to shed some light on what the software provides and give some practical recommendations on simple applications.

## Splines in a nutshell

The term ‘spline’ refers to a craftsman’s tool, a flexible thin strip of wood or metal, used to draft smooth curves. Several weights would be applied on various positions so the strip would bend according to their number and position. This would be forced to pass through a set of fixed points: metal pins, the ribs of a boat, etc. On a flat surface these were often weights with an attached hook and thus easy to manipulate. The shape of the bended material would naturally take the form of a spline curve. Similarly, splines are used in statistics in order to mathematically reproduce flexible shapes. Knots are placed at several places within the data range, to identify the points where adjacent functional pieces join each other. Instead of metal or wood stripes, smooth functional pieces (usually low-order polynomials) are chosen to fit the data between two consecutive knots. The type of polynomial and the number and placement of knots is what then defines the type of spline.

***Motivating example***

**Fig. 1:**
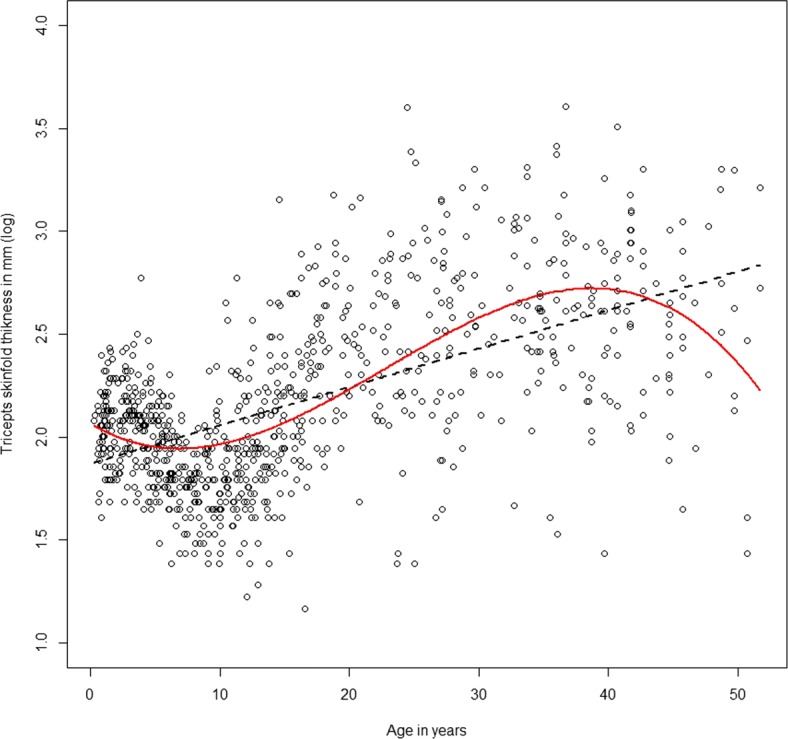
A plot of age in years against the triceps skinfold thickness for 892 females in West Africa [3, 23]. The dashed line represents a simple linear fit, the solid line a fit using flexible third degree polynomials

With the introduction of generalized additive models (GAMs) [15] in 1986, the use of spline modelling has become an established tool in statistical regression analysis. To illustrate this, consider data on a set of 892 females under 50 years collected in three villages in West Africa (data available in the Additional file 1: Appendix). We would like to explore the relationship between age (in years) and a crude measure of body fat, which is triceps skinfold thickness. Figure 1 shows the relationship between age and triceps skinfold thickness measured in logarithmic scale. For more information about the data see [3, 23].

A simple regression model of the form *y*_*i*_=*β*_0_+*β*_1_*x*_*i*_+*ε*,*i*=1,...,*n*, would hardly give an approximation of the observed pattern, since it is obvious that the relationship is not linear. The model can be extended to accommodate for non-linear effects using some polynomials. Then, non-linear effects could be modelled by a polynomial of degree 3 given by:

1 $$ y_{i}=\alpha_{0}+\alpha_{1} u_{i}+\alpha_{2} u_{i}^{2}+\alpha_{3} u_{i}^{3}+\epsilon  $$

where *u* is a function of *x* called *basis* function, defined here by:

$$U=\left[ \begin{array}{cccc} 1 & x_{1} & x_{1}^{2} & x_{1}^{3}\\ \vdots & \vdots & \vdots & \vdots \\ 1 & x_{n} & x_{n}^{2} & x_{n}^{3} \end{array}\right] $$

The regression model described in Eq. 1 is still a linear model, despite the fact that it provides a non-linear function of the predictor variable. The model is still linear in the coefficients and can be fitted using ordinary least squares methods. The basis can be created in R using function poly(x,3) with inputs *x* (referring to the variable), and *p* (referring to the degree of the polynomial). This leads to a simple univariate smooth model of the form: *y*_*i*_=*f*(*x*_*i*_)+*ε* where *f*() is some function/transformation of the predictor. Such a model can be easily fitted in R by using: lm(y ∼poly(x,3)). Despite the simplicity, polynomial regression has several drawbacks, the most important being non-locality. That means that the fitted function at a given value *x*_0_ depends on data values far from that point. It is easy to see this in action by fitting a polynomial to a set of data and moving one of the data points near the right edge up or down. As a result, the fitted function will usually change far from that *x* coordinate.

Consider, instead of fitting a global polynomial, partitioning the range of *x* into smaller intervals, utilising an arbitrary number and position of points, *τ*, also called the *knots*. A simple piecewise continuous model can be fitted by defining the functions: *f*_1_(*x*)=1,*f*_2_(*x*)=*x,f*_3_(*x*)=(*x*−*τ*_1_)_+_,*f*_4_(*x*)=(*x*−*τ*_2_)_+_,..., with “+” a function defined as:

$$u_{+}=\left\{ \begin{array}{cc} u, & \text{if}\, u>0\\ 0, & \text{if}\, u\leq 0 \end{array}\right. $$

The set of these functions lead to a composite function *f*(*x*).

### Definition of splines

The draftsman’s metal spline can assume arbitrary shapes, for instance, the cross-section of an airplane wing or the spiral of a centrifugal pump. For statistical applications we will assume curves of the form *f*(*X*), i.e., a single *y* value for each *x*. The predictor *x* can be a single variable or multiple variables. Our discussion will focus almost entirely on a univariate function with $X\in \mathbb {R}$. Define a set of knots *τ*_1_<...<*τ*_*K*_ in the range of *X*. A spline *f*(*X*) will be a smooth function, satisfying certain differentiability properties mentioned below, such that *f*(*X*) is a polynomial of degree *d*. Wooden or metal splines have continuous derivatives of all orders since they are a physical object. This is not true for statistical splines. Rather we impose a smoothness criterion that all derivatives of order less than *d* are continuous. A physical spline is linear beyond the last knot and we may impose a further constraint derivatives of order 2 or greater are zero at the leftmost and rightmost knots; splines with this additional constraint are known as “restricted” or “natural” splines. In order to obtain more flexible curves the number of knots or the degree of the polynomial can be increased. There is however a trade-off; increasing the number of knots may overfit the data and increase the variance, whilst decreasing the number of knots may result in a rigid and restrictive function that has more bias.

#### Representation by basis functions

Assume that the unknown function *f* is represented by a spline function with fixed knot sequence and fixed degree *d*. Because the latter functions form a vector space *V*, it is possible to write *f* as

2 $$ f(X)=\sum\limits_{k=1}^{K+d+1}\beta_{k} B_{k} (X) \,,  $$

where the *B*_*k*_ are a set of basis functions defining *V* and *β*_*k*_ are the associated spline coefficients. With *k* knots there are *k*+1 polynomials of degree *d* along with *d*∗*k* constraints, leading to (*d*+1)(*k*+1)−*d*∗*k*=*d*+*k*+1 free parameters [9, 41]; for a natural spline there are *k* free parameters. Since *βB*=(*βA*)(*A*^−1^*B*)=*γB*^∗^ for any nonsingular matrix *A* there are an infinite number of possible basis sets for the spline fit.

The representation in (2) has the advantage that the estimation of *f* reduces to the estimation of the coefficients *β*_*k*_. More specifically, the expression in (2) is linear in the coefficient vector *β*=(*β*_1_,...,*β*_*K*+*d*+1_). Therefore the estimation of *f* can be viewed as an optimization problem that is linear in the transformed variables *B*_1_(*X*),...,*B*_*K*+*d*+1_(*X*), allowing for the use of well-established estimation techniques for use of splines in a broad range of (generalized) multivariable regression models. Importantly, spline modelling reduces the estimation of the functions *f*() to the estimation of a small set of real-valued coefficients.

As pointed out by various authors (e.g., [9, 12, 41] the high flexibility of spline modelling comes at the price of a number of tuning parameters. Two of these, the choice of basis functions B and the degree *d* of the underlying polynomials turn out to have little impact. In fact, spline fits are remarkably robust to the degree *d*. Cubic polynomials (*d*=3) are the usual standard as they result in curves that appear perfectly smooth to the human eye. If derivatives of the fitted curves are of interest, a higher order is sometimes appropriate, but in general fits for *d*>3 are effectively indistinguishable. Fits with *d*=1 or *d*=2 have nearly identical statistical properties but will appear more jagged. The choice between two basis sets *B* and *B*^∗^ will by definition not change the predictions from a fit and so come down to convenience issues.

The two key choices are in the number and spacing of the knots and the use (or not) of a penalty function, e.g., the integrated second derivative of the spline. When there is no penalty, the creation of the transformed variables can be done separately and the new variables are simply included in a standard model fit; no modification of the underlying regression procedure is required. This approach is often referred to as *regression splines*; the flexibility of the resulting non-linear function is entirely a function of the number of knots. The inclusion of a smoothing penalty, on the other hand, requires modification of the fitting routine in order to accommodate it. This has to be included in each regression function separately. The resulting *smoothing splines* have several desirable properties, but the added complexity of the smooth function can be a reason for not been used more often in applied settings.

Although considerable research has been conducted to explore the mathematical properties of the various spline approaches (see [4, 11, 13, 37, 41], applied statisticians and data analysts hardly seem to be aware of these results when using spline modelling in practical applications. In fact, many of the articles identified by our web search contained no justification on the rationale for the choice of the used spline method.

### Popular spline basis

There are numerous options for the definition of the basis functions *B*_*k*_, where the various spline basis differ with respect to their numerical properties [4, 41]. In this Section, we will introduce some of the most popular spline basis, namely the truncated power series basis, the B-spline basis and the cardinal spline basis.

#### Truncated power series and Cubic splines

The truncated power series basis is defined by the basis functions

$$B_{1}(x) = 1, B_{2}(x) = x,..., B_{d+1}(x) = x^{d}, $$

$$B_{d+2}(x) = (x- \tau_{1})_{+}^{d},..., B_{K+d+1} = (x -\tau_{k})_{+}^{d} $$

**Fig. 2:**
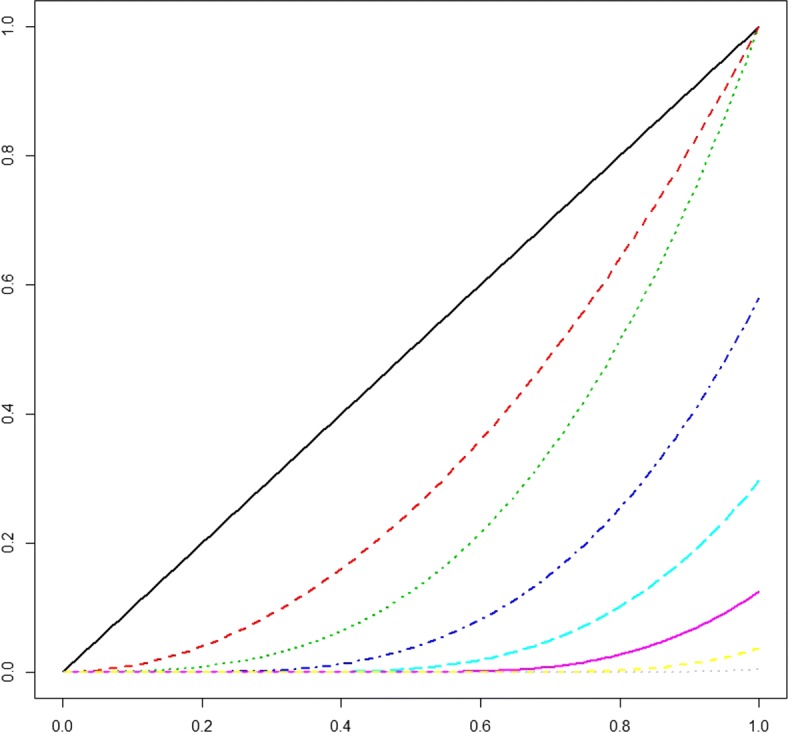
Truncated polynomials spline basis functions of third degree (*d*=3) with five equidistant knots (*K*=5). Plot created using Code *#*1 in the Additional file 1: Appendix

An advantage of the basis functions above is their easy interpretation: Starting with a “basic” polynomial of degree *d* defined on [*a,b*] (first line of equation), deviations from the basic polynomial are successively added to the spline function to the right of each of K knots (second line). A truncated power base spline is *d*−1 times differentiable at the knots and has *d*+*K* degrees of freedom. It is relatively easy for the user to create a truncated power series in R. Let *x* represent some observations in [0,1], then a truncated power basis of degree *d*=3 with 5 knots equally spaced within along the range of *x* can be created using Code 1 in the Additional file 1: Appendix (Fig. 2).

A feature of the truncated power series is that the supports of the functions are not local, with some of the *B*_*k*_ being defined over the whole range of data [*a,b*]. This might lead to high correlations between some basis splines, implying numerical instabilities in spline estimation. For the truncated power series basis, an example is given in [9], Chapter 5.

Cubic splines are created by using a cubic polynomial in an interval between two successive knots. The spline has four parameters on each of the *K*+1 regions minus three constraints for each knot, resulting in a *K*+4 degrees of freedom.

A cubic spline function, with three knots (*τ*_1_,*τ*_2_,*τ*_3_) will have 7 degrees of freedom. Using representation given in Eq. 2, the function can be written as:

$$ f(X)= \beta_{0} + \beta_{1} X + \beta_{2} X^{2} + \beta_{3} X^{3} + \beta_{4} (X-\tau_{1})^{3} + \beta_{5} (X-\tau_{2})^{3} + \beta_{6} (X-\tau_{3})^{3} $$

#### B-splines

The B-spline basis is a commonly used spline basis that is based on a special parametrisation of a cubic spline. The **B-spline basis** [4], is based on the knot sequence

$$\begin{aligned} \xi_{1} \le \ldots &\le \xi_{d} \le \xi_{d+1} < \xi_{d+2} < \ldots < \xi_{d + K + 1} \\ &< \xi_{d + K + 2} \le \xi_{d + K + 3} \le \ldots \le \xi_{2d + K + 2} \,, \end{aligned} $$

where the sets *ξ*_*d*+2_ := *τ*_1_,…,*ξ*_*d*+*K*+1_:=*τ*_*K*_ and *ξ*_*d*+1_:=*a*,*ξ*_*d*+*K*+2_:=*b* are referred to as “inner knots” and “boundary knots”, respectively. The choice of the additional knots *ξ*_1_,…,*ξ*_*d*_ and *ξ*_*d*+*K*+3_,…,*ξ*_2*d*+*K*+2_ is essentially arbitrary. A common strategy is to set them equal to the boundary knots. Alternatively, if the inner knots and the boundary knots *ξ*_*d*+1_<…<*ξ*_*d*+*K*+2_ are chosen to be equidistant, i.e., *ξ*_*k*+1_−*ξ*_*k*_=*δ* ∀*k*∈{*d*+1,…,*d*+*K*+1}, the boundary knots may be placed at *ξ*_*d*+1_−*δ*,…,*ξ*_*d*+1_−*d*·*δ* and *ξ*_*d*+*K*+2_+*δ*,…,*ξ*_*d*+*K*+2_+*d*·*δ*.

For *d*>0, B-spline basis functions of degree *d* (denoted by $B_{k}^{d}(x)$) are defined by the recursive formula^1^

$$ \begin{aligned} B_{k}^{d}(x)&=\frac{x-\xi_{k}}{\xi_{k+d}-\xi_{k}}B_{k}^{d-1}(x)-\frac{\xi_{k+d+1}-x}{\xi_{k+d+1}-\xi_{k+1}}B_{k+1}^{d-1}(x),\\k &= 1,...,K+d+1, \end{aligned} $$

where

$$B_{k}^{0}(x)=\left\{ \begin{array}{cc} 1, & \xi_{k} \leq x < \xi_{k+1}\\ 0, & \text{else} \end{array} \right. $$

and $B_{k}^{0}(x) \equiv 0$ if *ξ*_*k*_=*ξ*_*k*+1_. B-splines have the advantage that the basis functions have local support. More specifically, they are larger than zero in intervals spanned by *d*+2 knots and zero elsewhere. This property results in a high numerical stability, and also in an efficient algorithm for the construction of the basis functions, see [4] for details.

#### Natural cubic and cardinal splines

A polynomial spline such as a cubic or a B-spline, can be erratic at the boundaries of the data. To address this issue, natural splines are cubic splines that have the additional constraints that they are linear in the tails of the boundary knots (−*∞*,*a*],[*b*,+*∞*). This is achieved by requiring that the spline function *f* satisfies *f*^″^=*f*^‴^=0 which lead to additional four constraints, that a natural spline basis on *K* knots has *K*+1 degrees of freedom.

Another basis for natural cubic splines is the *cardinal spline basis*. The *K* basis functions of cardinal splines (of degree *d*=3 each) are defined by their values at the knots *τ*_1_,...,*τ*_*K*_. More specifically, they are defined such that the *k*-th basis function satisfies *B*_*k*_(*τ*_*k*_)=1 and *B*_*k*_(*τ*_*j*_)=0,*τ*_*j*_≠*τ*_*k*_. As a consequence, the coefficients *β*_*k*_ have an easy interpretation: Each coefficient equals to the value of the spline function *f* at the knot *τ*_*k*_. For an efficient construction of the cardinal spline basis we refer to [41], Chapter 4.

In addition to the truncated power series natural splines, B-spline and cardinal spline basis, various other - less popular - basis exist. For an overview, we refer to the books by [11, 13, 41].

### Penalized splines

The splines presented so far are often referred to as *regression splines*. In addition to the choice of the spline basis (B-spline, truncated power series, etc.), the number of knots and the knot positions have to be chosen. Obviously, these tuning parameters may have an important impact on the estimated shape of a spline function: A large number of knots implies high flexibility but may also result in overfitting the data at hand. Conversely, a small number of knots may result in an “oversmooth” estimate that is prone to under-fit bias (see [9, 41]).

A popular approach to facilitate the choice of the knot positions in spline modelling is the use of *penalized splines*. Given an i.i.d. sample of data (*x*_1_,*y*_1_),…(*x*_*n*_,*y*_*n*_), a penalized spline is the solution to the problem

$$\hat{\beta} = \text{argmax}_{\beta} \left[ l_{\beta} (x_{1},y_{1}, \ldots, x_{n},y_{n}) - \lambda \cdot J_{\beta} \right] \,, $$

where *l*_*β*_ denotes the log-likelihood (or, in case of Cox regression, the partial log-likelihood) and *J*_*r*_ is a roughness penalty that becomes small if the the spline function is “smooth”. Generally, penalized splines are based on the idea that the unknown function *f* is modeled by a spline with a large number of knots, allowing for a high degree of flexibility. On the other hand, a rough spline estimate that has a high value of *l*_*β*_ and is close to the data values results in a large value of *J*_*β*_. The maximization of this function therefore implies a trade-off between smoothness and model fit that is controlled by the tuning parameter *λ*≥0.

A special case is the penalized least squares problem

3 $$ \hat{\beta} = \text{argmin}_{\beta} \left[ \sum\limits_{i=1}^{n} \left(f_{\beta} (x_{i}) - y_{i}\right)^{2} + \lambda \cdot {\int\nolimits}_{a}^{b} \left(\partial^{2} f / \partial x^{2}\right)^{2} \,dx \right]  $$

in Gaussian regression. The penalty $J_{\beta } \,=\, \int _{a}^{b} \left (\partial ^{2} f / \partial x^{2}\right)^{2} dx$ expresses the “smoothness” of a spline function in terms of the second derivative of *f*. For given *λ*, it can be shown that the solution is a natural cubic spline with knot sequence *x*_(1)_<…<*x*_(*n*)_, i.e., the knot positions do not have to be chosen but are ‘naturally’ given by the ordered unique data values of *X*. In the literature, this type of spline is referred to as *smoothing spline* [11]. Of note, it can be shown that a smoothing spline interpolates the data if *λ*=0, while *λ*=*∞* implies a linear function. Note that smoothing splines are a special case of the more general class of *thin plate splines* [40], which allow for an extension of the criterion in Eq. (3) to higher-dimensional *x*_*i*_ (see [41], Section 4.15], and [11] for details).

A convenient property of smoothing splines is that the penalty *J*_*β*_ can be written as *β*^⊤^*Ωβ* with a suitably defined penalty matrix *Ω*. Therefore the solution to (3) is given by the penalized least squares estimate

4 $$ \hat{\beta} = \left(B^{\top} B + \lambda \Omega\right)^{-1} B^{\top} y  $$

where *B* is a matrix of dimension *n*×*n* containing the natural spline basis functions evaluated at the data values. The vector *y* contains the response values *y*_1_,…,*y*_*n*_. In practice, very efficient algorithms exist to compute $\hat {\beta }$ in (4) [11]. Instead of specifying a natural spline basis for *f*, it is further possible to work with an unconstrained B-spline basis, as the penalty in (3) automatically imposes the linearity constraints at the knots *x*_(1)_ and *x*_(*n*)_ (see [9], Chapter 5, and [13], Chapter 2). Regarding the B-spline basis, estimation results will not depend on the choice of the boundary knots:it is either possible to use *x*_(1)_ and *x*_(*n*)_ as boundary knots or to include *x*_(1)_ and *x*_(*n*)_ in the set of inner knots.

If *n* is large and the interval [*a,b*] is covered densely by the observed data, it is usually not necessary to place a knot at every *x*_*i*_,*i*=1,…,*n*. Instead, the smoothing spline may be approximated by a *penalized regression spline* that uses a reduced set of knots. A very popular class of penalized regression splines are *P-splines* [8], which are based on the cubic B-spline basis and on a ‘large’ set of equidistant knots (usually, 10–40). Instead of evaluating the integral in (3), P-splines are based on a second-order difference penalty defined by

$$J^{*}_{\beta} = \sum\limits_{k=3}^{K+4} \left(\Delta^{2} \beta_{k} \right)^{2} \,, $$

which, in case of evenly spaced knots, can be shown to be an approximation to *J*_*β*_. The second-order difference operator *Δ*^2^ is defined by *Δ*^2^*β*_*k*_:=(*β*_*k*_−*β*_*k*−1_)−(*β*_*k*−1_−*β*_*k*−2_). The penalty can therefore be expressed as *β*^⊤^*Pβ*, where *P* is defined by *D*^⊤^*D* with *D* a matrix of differences. It is easily derived that the resulting estimator of *β* has the same structure as 2, with *Ω* replaced by *P*.

A convenient property of P-splines is that they are numerically stable and very easy to define and implement. In particular, it is much easier to set up the difference matrix *D* than the matrix *Ω*. Also, it is straightforward to extend the penalty *J*_*β*_ (and hence the matrix *D*) to higher-order differences *Δ*^*q*^ with *q*>2. It is also possible to use a knot sequence that is not evenly spaced; in this case, weights need to be introduced. Because P-splines with unevenly spaced knots are seldom used in practice, we do not consider them here and refer to [8] instead.

Smoothing splines and P-splines overcome the problem of knot selection to some degree. Their philosophy is to use a large number of knots and then let *λ* control the amount of smoothness. This results in one extra tuning parameter, with no general consensus on how to tune this parameter. Some popular ways to determine the “optimal” value of *λ* use generalized cross-validation (GCV), AIC or a mixed-model representation [24].

### Splines in R

The basic installation bundle of R contains a set of functions that can fit simple polynomial splines and smoothing splines. Further functions are included in the library splines written by DM Bates and WN Venables. The package has been the workhorse of spline fitting for many years and is now part of the basic distribution of R. There are more than 100 other packages that depend on splines when loading. The package contains several functions to create spline basis, such as bs for B-splines and ns for natural splines, that are widely used, but also some more specialized functions for creating basis functions (such as periodicSpline that creates a periodic interpolation splines) or commands that are useful such as command predict.bSpline that would evaluate a spline at new values of *X*.

**Fig. 3:**
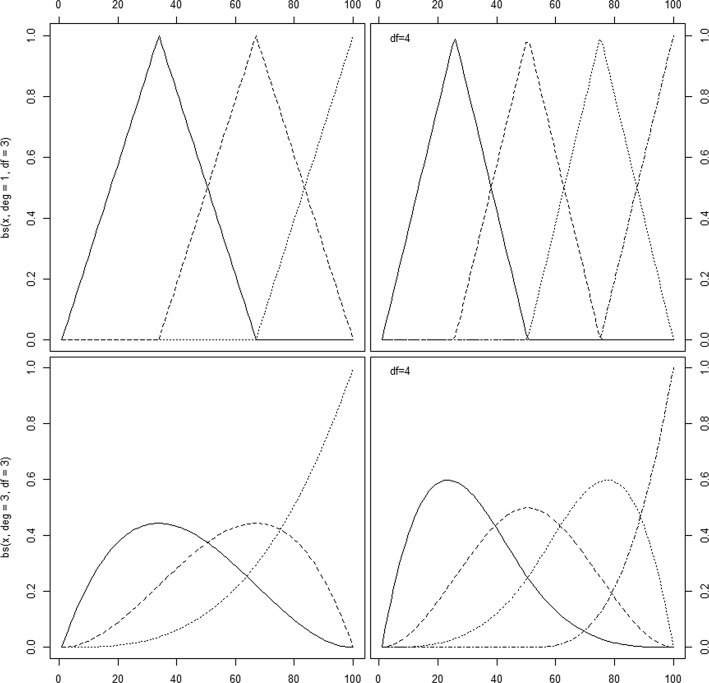
B-spline basis using bs command in library splines. Top left: Spline basis of first degree with three degrees of freedom. Top right: Spline basis of first degree with four degrees of freedom. Bottom left: Cubic spline basis with three degrees of freedom. Bottom right: Cubic spline basis with four degrees of freedom. Graphs created using Code *#*2

The default bs values will create a cubic B-spline basis with two boundary knots and one interior knot placed at the median of the observed data values. More flexibility can be achieved by the user, by increasing the placement and the number of knots and/or changing their locations. Figure 3 (code 2 in the Additional file 1: Appendix) shows B-splines created with different options. The upper part presents linear splines, i.e. first order polynomials (degree is one) connected together on equidistant knots. The lower part presents cubic polynomials (degree 3).

It should be noted that B-splines created in R with bs() are automatically bounded by the range of the data, and that the additional knots (*τ*_1_,...,*τ*_*d*_) are set equal to the boundary knots, giving multiple knots at both ends of the domain. This approach is useful in univariate cases and has some computationally attractive features. However, if one works on a two-dimensional smoothing problem, using tensor products of B-splines, or when working with P-splines, this basis is unsuitable and may lead to spurious results.

**Fig. 4:**
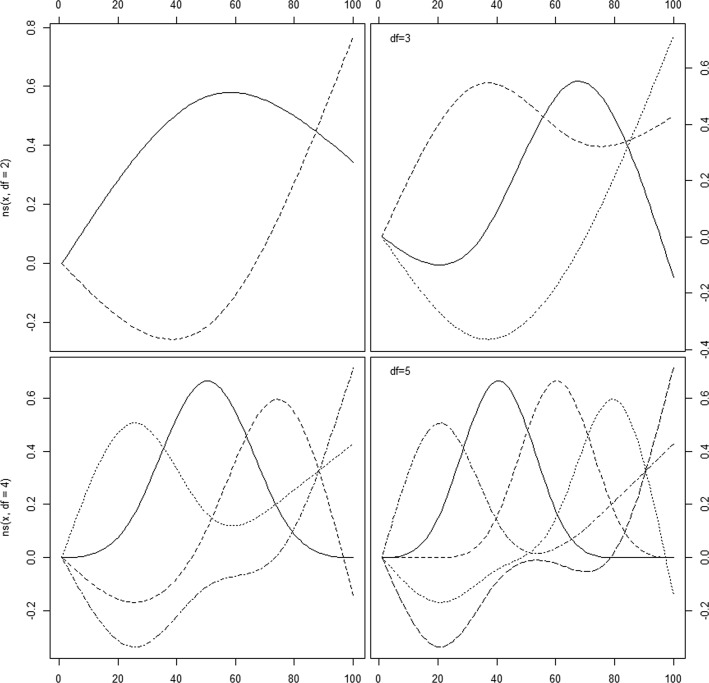
Natural cubic spline basis using command ns in library splines. Top left: Spline basis with two degrees of freedom. Top right: Spline basis with three degrees of freedom. Bottom left: Spline basis with four degrees of freedom. Bottom right: Spline basis with five degrees of freedom. Created with Code#3

Natural splines can be created within the splines package, using command ns. By default, unless the user specifies either the degrees of freedom or the knots the function returns a straight line within the boundary knots. Figure 4 (code 3 in the Additional file 1: Appendix shows natural splines created with different options.

**Fig. 5:**
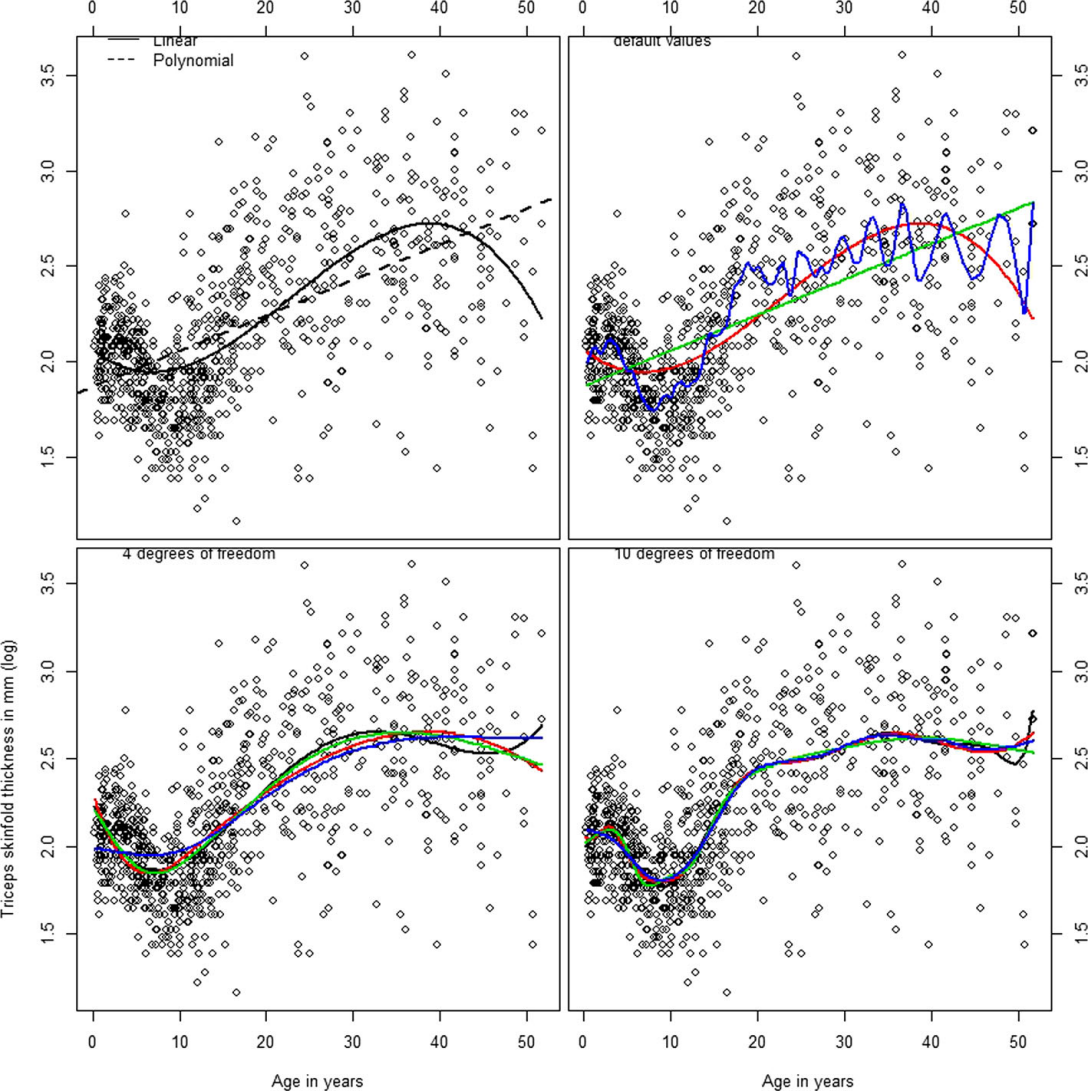
A plot of age in years against the triceps skinfold thickness for 892 females in West Africa. Upper left: Dashed line represents a simple linear fit, solid line a fit using flexible third degree polynomials. Upper right: Splines fit using default R values. Green line is the result of a polynomial spline of degree 1 (default value for function poly, and a fit from a natural spline with no degrees of freedom specified (default value for functions ns). Red line comes from a b-spline with three degrees of freedom (function bs and blue line from a smoothing spline (from function smooth.spline). Lower left: Black line is polynomial fit, red line b-splines fit, green line is a natural splines fit and smoothing spline, all defined with four degrees of freedom. Lower Right: Same functions defined with 10 degrees of freedom. Created with Code #4

To illustrate how these functions can be used in practice, consider again the data from Section 2.0.1. Figure 5 (created by (code 4 in the Additional file 1: Appendix)) shows the fits obtained by using the following commands: poly() for simple orthogonal polynomical splines, smooth.spline() for smoothing splines, bs() and ns() from library splines, for B-splines and natural splines respectively. The upper left graph shows a simple linear fit on the data (dashed line) and a third degree polynomial fit that is able to capture the more complex relationship between the variables. The graph on the upper right corner is particularly interesting though, since it presents the fits using the default values of the spline functions. The green line comes from functions poly() and ns() which at default they both define a straight line. On the other extreme, the blue line is a fit from function smooth.spline() which if no degrees of freedom are specified tends to undersmooth the data, i.e. produce a very flexible wiggly fit based -here- on 45 degrees on freedom. A -visually- reasonable fit to the data can be achieved when four degrees of freedom are specified (lower left graph). It can be seen that there are some differences depending on the chosen base. The polynomial basis (black line) is a little more flexible than the rest, especially at higher ages. On the other hand, a smoothing spline restricted to just four degrees of freedom is more rigid than other approaches, but probably oversmooths the data at small ages, between years 0 and 10. In between the two extremes, B-splines and natural splines provide very similar fits that capture the effect of small ages and tend to be less influenced by extreme cases at the end of the age spectrum. Last, the lower right graph shows how much more flexible the fits become with additional degrees of freedom and suggests potential over-fit bias due to use of excessive degrees of freedom.

#### A note on degrees of freedom

In practice, it is always useful to define a spline by degrees of freedom. This approach is particularly useful when working with B-splines and natural splines. B-splines have *d*+*K*, while a natural cubic spline basis function with *K* knots has *K*+1 degrees of freedom, respectively. By default, the function bs in R creates B-splines of degree 3 with no interior knots and boundary knots defined at the range of the *X* variable. As such the function creates three basis functions. Now consider the following case: when a user defines a B-spline with an interior knot at the median of *X* (bs(x,knots=median(x))) the software will create four functions (*d*=3 plus *K*=1 interior knots, four degrees of freedom). If however, the user specifies in the function the boundary knots within the knots argument (bs(x,knots=c(min(x),median(x),max(x)))), the function will have six degrees of freedom (d =3 plus k =3). Similar caution should be taken with function ns.

When working with smoothing splines, it is not easy to specify the degrees of freedom, since they will vary depending on the size of the penalty. However, in practice, penalized splines can also be restricted to a maximum number of degrees of freedom or desired degrees of freedom.

#### Other spline packages

Broadly speaking, the extended list spline packages contains either approaches that are quite similar to what is presented here or very specialized cases that target specific applications. In Table 1 some of these packages are presented along with the number of downloads. The number refer to the number of times a package has been downloaded but not unique users. It is beyond the scope of this work to describe in detail all of these approaches.

**Table 1:** R packages used for the creation of splines

Package	Downloaded	RD	Description	Authors
gss	632212	9	General smoothing splines	Chong Gu
rms	598185	63	Regression modeling strategies	Frank Harrell Jr
polspline	406661	11	Polynomial spline routines	Charles Kooperberg
pspline	146939	11	Penalized smoothing splines	Brian Ripley
logspline	130048	10	Logspline density estimation routines	Charles Kooperberg
cobs	58533	6	Constrained B-splines	PT Ng and M Maechler
crs	58347	2	Categorical regression splines	JS Racine, Z Nie, BD Ripley
splines2	31031	4	Regression spline functions and classes	Wenjie Wang and Jun Yan
bigsplines	25940	1	Smoothing splines for large samples	Nathaniel E. Helwig
bezier	18483	1	Bezier curve and spline toolkit	Aaron Olsen
pbs	17794	1	Periodic B splines	Shuangcai Wang
freeknotsplines	13761	0	Free-knot splines	S Spiriti, P Smith, P Lecuyer
orthogonalsplinebasis	13436	1	Orthogonal B-spline functions	Andrew Redd
ConSpline	10565	0	Partial linear least-squares regression using constrained splines	Mary Meyer
episplineDensity	9375	0	Density estimation exponential	S Buttrey, J Royset, R Wets

The number of times of time each package was downloaded is measured from 01/10/2012 to 15/11/2018. Number of downloads does not correspond to unique users. Reverse dependencies (RD) stands for the number of other packages that call each one

## Regression packages

***The general idea of regression with splines***

A regression model, in which splines are used to model the effects of continuous variable(s) is a special case of multivariable regression, where some ’predictors’ are non-linear functions of the original explanatory variable(s). Here, we consider spline modelling in the context of regression type models predominant in medical research, such as Gaussian regression, logistic and counts regression or time to event regression. A general (main-effects) representation of these models (quantifying the effects of some explanatory variables *X*=(*X*_1_,...,*X*_*p*_) on an outcome variable) can be written as

$$g(Y) = \beta_{0} + f_{1}(X_1) +... + f_{p}(X_{p}) $$

where *g*(.) is the link function and the unknown functions *f*_1_,...,*f*_*p*_ are estimated from the sample data. In case of a continuous explanatory variable *X*_*j*_,*j*∈1,...,*p*, the function *f*_*j*_ may have a linear or arbitrary non-linear shape and is assumed to be smooth, and spline modelling constitutes a highly flexible approach to estimate *f*_*j*_. In fact, since each spline function can be written as a linear combination of a set of pre-defined basis functions, parameter estimation relies on established approaches for linear predictors, and a number of efficient algorithms for spline fitting exist [13, 41]. While we restrict our analysis to the main-effects model above, it should be emphasized that spline modelling also allows for incorporating interaction terms between covariates. For example, a two-way non-linear interaction surface of the form *f*_*jk*_(*X*_*j*_,*X*_*k*_) could be modelled using tensor product splines. For an in-depth discussion of interaction modelling using splines see, in particular, Harrell [12] and Wood [41]. However, in this article, we will restrict our analysis to the main effects.

***The packages***

There are several packages that can fit regression models using some sort of splines available in R. For the purposes of this review, only a handful of packages has been selected, with focus on packages that deal with methods usually used in the analysis of observational studies. All of the chosen libraries focus on linear and generalised linear models, generalised additive models or smoothing methods and have been downloaded a substantial number of times (See Table 2). Furthermore, the packages come with several help files, vignettes, books or website supplements to guide the user through their use and include real life data, clear references and a wide range of examples so it is easier to evaluate their quality. The selected packages are presented in Table 2 which also includes a short description of what the packages do.

**Table 2:** Regression packages selected for further analysis

Name	Description	Author
gam	Generalized additive models	T. Hastie
mgcv	Mixed GAM computation vehicle with GCV/AIC/REML	S. Wood
VGAM	Vector generalized linear and additive models	T.W. Yee
gamlss	Generalised additive models for location scale and shape	M. Stasinopoulos

The gam library [14] is one of the main packages that can be used for fitting and working with Generalized additive models, as described in Chapter 7 of [2], and [13]. The package contains code that fits several different generalized regression models, with several different types of responses (see Table 3). The package requires splines when is loaded for fitting additive models.

**Table 3:** General features of popular regression packages

Package	Downloads	Vignette	Book	Website	Datasets
quantreg	5099669	X	X		8
survival	3511997	X	X		38
mgcv	3217720	X	X		2
gbm	668984			X	0
VGAM	662399	X	X	X	50
gam	459497		X	X	4
gamlss	210761	X	X	X	43

Downloads refer to the number of times a package has been downloaded (starting from 26-06-2013 up to 15-11-2018), not unique users. Also general information about whether the package has a vignette, a book or a website. Last column presents the number of real datasets available with the package

Using download numbers as a criterion the most popular package in the list is mgcv [39]. The package is particularly useful for fitting spline models, and it includes many functions that perform smoothness estimation, fit generalized additive and mixed models. Part of the popularity of the model can be explained by the fact that it requires minimum input from the user when working with splines. This feature sometimes might allow researchers to apply a sophisticated model, but quite often it is difficult for an applied user to understand how exactly the smoothing terms are estimated and what are the theoretical implications of the approach. The code performs smoothing parameter estimation by automatically using generalized cross validation or other advanced statistical methods. It is also quite well documented with several vignettes and examples available at the author’s website, and in the companion book [41]. What also distinguishes the package from the rest, is that it does not require splines to create the spline basis. Instead, all basis are created within the package itself, with the default being thin plate regression splines [40].

Another powerful package VGAM [42] was created by TW Yee for fitting vector generalized additive and linear models [43]. The package is quite powerful, in the sense that can fit a range of complicated statistical methods, including multivariable GLMs, non-linear and reduced rank models amongst other. In fact, the merit of the software can be seen in these advanced modelling approaches. When used in a simpler setting, the package boils down to using the same approaches as gam. Package gamlss [20] contains functions for fitting, displaying and checking generalised additive models for location scale and shape (GAMLSS) [31]. This family of models extends on generalized linear and additive models and the package contains numerous useful tools for spline fitting, including P-splines and two-dimensional smoothing.

It has to be stressed that packages that were not included to the list can be of great importance or significance but may not fall within the scope of this work. For instance, even though quantreg [17] is a package that has a large number of downloads, quantile regression is not a commonly used technique in the analysis of medical data, yet. Boosting [28] is also of interest but due to limited space, and the fact that it is based on a different modelling approach, relevant packages are not discussed here. Last, package survival [34] was also left out solely because it is only designed to fit survival models and is therefore difficult to compare it to more generic regression packages. All of these packages are powerful and can fit several different models. Moreover, all of these packages include functions to fit P-splines, and most of them (with the exception of gam) can include random effects.

***Working with regression packages***

**Fig. 6:**
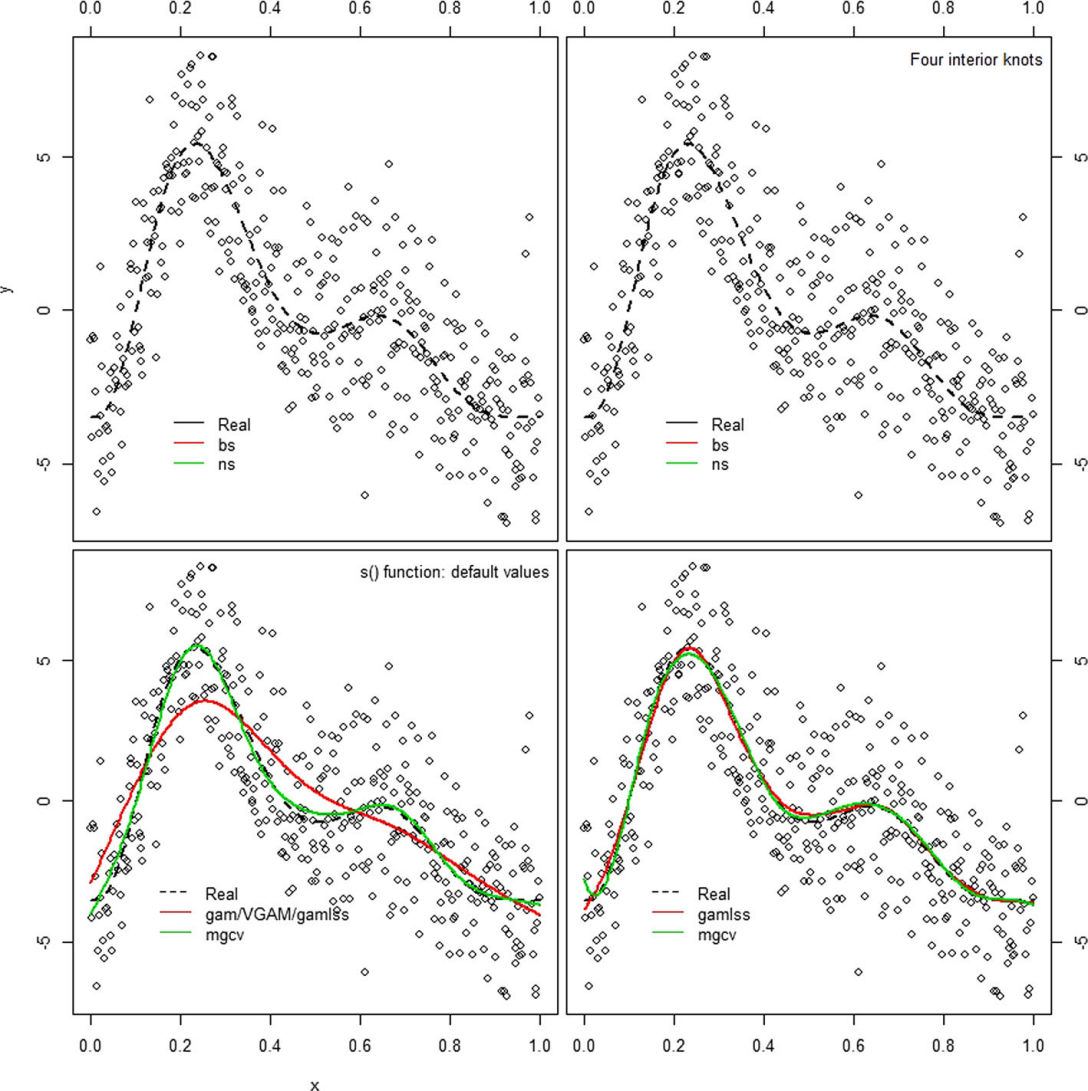
Scatter plot of simulated data points with different spline fits from packages gam, mgcv and gamlss. Upper left: Data were fitted with library gam that calls B-spline and natural spline functions from splines package. A B-spline with 3 degrees of freedom is the default bs value. Natural splines were used also with three degrees of freedom. The two basis are different, especially in the tails of the x distribution. It is apparent that more flexibility is needed to approach the true curve, given by the dashed line. Upper right: Data fitted with library gam, with added flexibility. Both B-splines and natural splines were defined with four interior knots, resulting in a B-spline with 7 degrees of freedom and a less flexible natural spline with 5 degrees of freedom. Lower left: Comparison of data fitting at default values using function s, in packages mgcv, gam and gamlss. The thin plate regression splines are more flexible than the cubic smoothing spline used by gam and gamlss. Lower right: Comparison of data fitting at default values using P-splines. The differences are rather small and can be attributed to the different way that two packages optimize the penalty weight. Created with Code #6

A simple simulation study is used to illustrate the use of the packages. Let *X* be a sequence of *n*=400 values uniformly distributed in (0,1). The response is then generated as

$$ y \,=\, -3.5 + 0.2 \times X^{11} \times 10 \times (1 - X)^{6} + 10 \times \left(10 \times X^{3}\right) \times (1- X)^{10} +\epsilon $$

where the errors *ε* are simulated from a normal distribution with mean zero and standard deviation *σ*=2. Figure 6 (upper) illustrates how these data that are designed to have a flexible pattern. In order for a model to follow such a pattern, splines would require added flexibility, which means more degrees of freedom. Packages gam, gamlss and VGAM call on the basic splines package to create B-splines and natural splines. As such, the code to create a spline fit with either basis would be almost identical and would result in the same fit to the data. However, when used, the summary given for the object created is different (see Code#5 and Code#6 in the Additional file 1: Appendix. In mgcv B-splines and natural splines can be fitted by using the s function, analysed in the next section.

***The s function***

It is common practice in many R regression packages to use an s function when defining the formula of a model. The function is a symbolic wrapper used to indicate a smooth term in the model. Depending on the package, the function then calls the appropriate function to create the basis and model matrix, or terms of the model. Although the code may look similar, or in many cases identical, different packages use different default values and refer to other basis functions when applying a model. The function is common in gam, VGAM and mgcv.

Both packages gam and VGAM call the function smooth.spline with four degrees of freedom as default and give identical results. Under gam package the model would be specified as: gam(y s(x)), while VGAM would fit the same model with vgam(y s(x), family=gaussianff). In gamlss, the s function is not available. Instead, the user has to specify cs if a cubic smoothing spline is needed, using command line: gamlss(y cs(x)).

When working with mgcv, although the command line is gam(y s(x)), identical to gam, the package creates by default thin plate regression splines. The user has the choice to define the maximum degrees of freedom (by default these are set to 10) or how the penalty is maximized (by default, generalized cross validation is used). Other spline types can be defined as well, including B-splines, cubic splines and more. In the bottom left panel of Fig. 3 the results of fitting different models at default values using the s function, are presented. mgcv uses its own code to produce a flexible (green) curve that follows quite well the simulated values of y data.

***Working with P-splines***

Penalised splines can be a great tool to describe complex non-linear relationships. Marx and Eilers [7, 8] argued that researchers should not worry about the amount and placement of the knots, instead: they used a great number of knots and let the fit to be controlled by a penalty. Both mgcv and gamlss include P-splines and an automated way to optimize the penalty weight. In mgcv, the option ps within the s function will create a cubic spline basis on a default of 10 knots, with a third order difference penalty. The penalty weight is optimized with generalized cross validation. Users can change these options and define the Un-biased Risk Estimator [10] or AIC criteria for penalty optimization. When working with gamlss, the function pb defines cubic B-splines functions with 20 interior knots and a second order difference penalty. The smoothing parameter is estimated using local maximum likelihood method (described in [19], and [18]) but there are also other options based on likelihood methods, AIC, generalized cross validation and more. For details refer to [31]. These approaches create a similar fit, as it can be seen in the lower right graph of Fig. 6. the two curves presented in the graph are created using a different number of knots (10 in mgcv vs 20 in gamlss), different order of penalty differences and a different way to optimize the penalty weight. However, the differences are rather small. That illustrates the merit of P-splines, where the penalties are very powerful in controlling the fit, given that enough knots are supplied into the function.

## Discussion

The project investigated all R packages that could be used for fitting splines in regression setting. We now have a better understanding of the field, the rate with which R packages appear and their general scope. To be consistent with the aims of STRATOS we had to narrow down the analysis to a few packages that would be useful to experienced analysts with little knowledge on this particular field. Analysts with low level of statistical knowledge will need much guidance before being able to use these powerful approaches for a better modelling of continuous variables. In a follower paper we will discuss and illustrate key issues of promising approaches and will compare derived functions and models in several examples.

Although we restricted our examples to linear and generalised linear models, all of the methods presented in this work could be used in the framework of survival analysis. The basic principles of the definition/construction of splines (bases, number and placement of knots etc.) are, in theory, independent of the type of outcome, and will therefore also work for time-to-event models with censored outcome and additive predictors. The same applies to penalization strategies (including the definition of the penalties, e.g., in P-splines) will also work for time-to-event models. For example, in Cox regression, P-splines can be incorporated and modelled by replacing the least squares criterion in Equation 3 by the partial log-likelihood. Analogously, in Weibull or log-normal survival models, one could insert a respective log-likelihood. Several examples of modelling survival data with splines can be found in [35] (chapter 5). The survival package has evolved from the S version [34] and is one of the most well documented libraries available in R. Still, we intend to work more on the use of splines for semiparametric analysis of interval-censored survival, competing risks and multistate process data in medical research. An overview of such regression packages and in depth evaluation will need further work in a follow up project.

Splines were reviewed having in mind two major families: regression splines and smoothing splines. The advantage of regression splines has to be simplicity: most of these can be fitted without even the need to go into a specialized package. We focused on B-splines and natural cubic splines since these are the ones that are included in the splines package but also are some of the most basic and popular choices in biomedical research. Smoothing splines can be more difficult to apply and understand, since the penalty term is not intuitively understood. However, they offer advanced flexibility and can be extremely helpful to identify complex patterns, without the need for the user to specify a number of parameters.

We presented a small overview of spline methods and just a few of the R packages that may be utilised for spline fitting and commended on their use. The review is far from extensive. The sheer volume of R packages that are created and uploaded on the web makes the task of reviewing all software rather daunting, but also irrelevant. Many of these packages will never reach a broad audience. While the present paper presented an overview of packages, we restrained weighting the presentation. The reason for this is that it is rather difficult to objectively judge how popular an R package actually is. For example, while download numbers can be seen as an indicator of popularity, these may be biased by inclusion of a package in pre-packaged distributions. Other potential indicators, such as whether a package receives regular feature updates or bug fixes, are even more difficult to handle. In the end, there might be a much coarser criterion, whether or not a package is part of the standard R distribution, that determined relevance for discussion. Yet, this would leave only few packages, and the “mgcv” package as the sole multivariable approach, potentially missing a lot of the opportunities brought by the plethora of available splines packages. Subsequent research will need to investigate how much is really gained by deviating from the standard distribution path.

One of the aims of this work is to come up with some practical recommendations. This paper has reviewed a number of packages in order to broaden our understanding of the field. We still need to work in more detailed comparisons, using simulated data and more complex datasets in order to come up with detailed recommendations and a thorough comparison of methods. For the time being we looked into more detail a selection of packages, including library splines for creating spline functions, and mgcv or gamlss for regression modelling. Library gam was also included in the text, mainly for historic reasons. This was one of the first libraries that gave the functionality to fit additive models that was based on previous functions written in S language. Many older users that migrated from S into R would have found the package very helpful and many would probably still use it today. The package incorporated spline smoothing with the requirement of splines package, but also has some useful functions to display the fitted functions. However, more modern packages have more functions and procedures to help the users.

Mixed GAM Computation Vehicle with Automatic Smoothness Estimation, or mgcv is the package that offers many possibilities, has a large number of downloads and is currently supplied with the basic distribution of R. The package includes many different spline basis: thin plate regression splines, cubic regression splines and cubic regression splines with shrinkage, cyclic cubic regression splines and p-splines. All of these basis are relatively easy to use with a specification in the s fuction. The package performs automatic estimation of the smoothing terms and that makes it particularly useful in practice. It is important that the package is well documented and the help files provide enough details for the user to understand what is hidden behind the code. The package comes with several online material and a very well written book. There are just two sample datasets in mgcv, but the required nlme package also loads 41 datasets that can be used for better understanding of procedures. The added functionality of mgcv includes smoothing in two dimensions, allows the users to specify their own spline basis and also contains procedures for variable selection.

A second package that was presented here is used to fit Generalised Additive Models for Scale and Location, gamlss. The package is not as popular as mgcv in download numbers but it does offer a wide variety of options and functions. The package contains functions to fit polynomials and piecewise polynomials, B-splines and P-splines, cubic splines, thin plate splines, monotonic smooth splines, cyclic smooth splines as well as functions for fractional polynomials. It also includes functions for smoothing in two dimensions, and other smoothers based on neural networks, varying coefficient models and others. Automatic selection of smoothing parameters can be performed, as well as variable selection. A great advantage of the package is that it has several other accompanying packages that include data and demos of how to fit gamlss within R. These packages along with well written help files, a number of online vignettes, a website and a book contribute into making the modelling methods more accessible to applied researchers. The gamlss.demo package contain functions to demonstrate some of the methods and can be a useful tool for teaching statistics. gamlss also has extension packages that can be used for boosting methods or censored data.

In the examples presented there were differences between the different approaches. These differences illustrate the challenges that an analyst faces when working with data, since most of the differences can be attributed to the choice of parameters rather than basis or approach used. In fact an experienced user will know how to obtain a reasonable outcome, regardless of the spline used. In practice, the different fits will have to do more with the degrees of freedom of the spline rather than the basis itself.

It is expected that many users will probably use a function at the default values of the software. However, using off-the-self software has been documented to lead to problems [6]. Therefore, it is important that these values are sensible and provide reasonable results in ‘common’ situations. Both mgcv and gamlss use appropriate default values that should provide a reasonable fit in most situations. However, we aim to scrutinize these packages in a follow up work and see how they perform in a variety of situations. Suitable default values are a good starting point for many analyses, but it is obvious that the specific aim of a study has an important influence on the usefulness of a model and the answer to the question whether a simpler or more complex model is preferable.Potential problems caused by underfitting and overfitting are assessed differently and a suitable model for prediction may be less useful as a model for explanation [29].

Accordingly, the choice of model selection criteria should balance the competing objectives of conformity to the data and parsimony [1, 26]. This issue is closely related to the selection of a simpler or more complex regression model, for example by preferring AIC or BIC as the criterion for variable selection. Consequently, the default values of a spline package can be a very good choice to derive a model for one aim and a bad choice for another aim. These issues are more discussed in the context of variable selection, model complexity and model stability, but they transfer to the choice of functions for continuous variables. This is discussed and illustrated in some example datasets analysed with the multivariable fractional polynomial (MFP) and spline based approaches [27]. It is obvious that the analyst cannot rely on the default but needs to know about the role of key parameters for selecting a suitable spline.

## Conclusions

Compiling guidance documentation for the use of splines is not an easy task. This review shows that it is very difficult for researchers to keep up with the sheer volume of new software packages and and even a group of experienced researchers is not able to critically assess and evaluate their quality. Instead of providing a review of all available software we emphasised on a subset of commonly used R packages that are well established in the field of biostatistics.

Futhermore, our work illustrates the challenges that analysts face when working on their own data. Experienced users and experts in the field of smoothing may be able to obtain reasonable outcomes in a variety of cases and regardless of the spline basis used. In these limited examples presented here, most differences can be attributed to the choice of hyper-parameters, rather than the basis used. In practice though, many researchers may choose to use software off-the-shelve, a strategy which carries many dangers. We need to accept the fact that many analysts do not have sufficient knowledge to use powerful tools adequately. The STRATOS initiative aims to provide more guidance and in depth comparisons of methods in future work.

## Additional file

Additional file 1 Appendix: R code. (PDF 57 kb)

## Abbreviations

CRAN: Comprehensive R Archive Network
GAM: Generalised Additive Model
STRATOS: STRengthening Analytical Thinking for Observational Studies

## Footnotes

1 Eilers and Marx [7] provide a simpler algorithm to obtain B-splines, based on a difference algorithm.
