# Supplementary material for: A review of spline function procedures in R
Source: BMC Med Res Methodol. 2019 Mar 6;19:46. doi: 10.1186/s12874-019-0666-3 (PMC6402144; doi:10.1186/s12874-019-0666-3)
Supplement: Supplementary file 1 — Appendix: R code. (PDF 57 kb) [file 12874_2019_666_MOESM1_ESM.pdf]

Appendix

Code 1

```
U <- seq(0, 1, length=100)
D <- 3  # degree of series
K <- 5  # number of knots
knots <- (1:K) / (K+1)  # creates a series of K equidistant knots
X1 <- outer (U, 1:D, "^") #a s in eq.2
X2 <- outer (U, knots,">") * outer (U, knots, "-")^D # as in eq.3
Bt <- cbind (X1, X2)
matplot(U, Bt, 'l', lwd=2, col= 1:ncol(Bt))
```

Code 2

```
x <- seq(0, 1, length=100)
library(splines)
par(mfrow= c(2, 2))
matplot(bs(x, degree=1, knots=c(0,.5,1)), type='l', main= "Degree=1")
matplot(bs(x, degree=2, knots=c(0,.5,1)), type='l', main= "Degree=2")
matplot(bs(x, degree=1, knots=c(0,.25,.5,1)), type='l', main= "Degree=1")
matplot(bs(x, degree=3, knots=c(0,.25,.5,1)), type='l', main= "Degree=3")
```

Code 3

```
par(mfrow= c(2, 2))
matplot(ns(x,df=2), ylab= "", type='l', main="Df=2")
matplot(ns(x,df=3), ylab= "", type='l', main="Df=3")
matplot(ns(x,df=4), ylab= "", type='l', main="Df=4")
matplot(ns(x,df=5), ylab= "", type='l', main="Df=5")
```

Code 4

```
## function to define spline basis
pbase <- function(x, p) {
u <- (x - min(x)) / (max(x) - min(x))
u <- 2 * (u - 0.5)
P <- outer(u, seq(0, p, by = 1), "^")
P
}
## Data available in http://mfp.imbi.uni-freiburg.de/book
## load data into R session and assign x <- age ; y <- lntriceps

## define plotting parameters
vdist <- hdist <- 0.2
layout( matrix(1:4, 2, 2, byrow=TRUE), widths=c(10,10), heights=c(10, 10))
par(mar= c(vdist, 4, 3, hdist))
```

```
plot(x, y, ylab="", xlab="Age in years", axes=FALSE)
axis(2); axis(3); box()
abline(lm(y~x), lwd=2, lty=2)
U <- pbase(x,3)
lines(x, U %*% coef(lm(y~U-1)), lwd=2)
legend(0.05, 3.8, c("Linear", "Polynomial"), col=1, lty=1:2, bty="n")
par(mar= c(vdist, hdist, 3, 4))
plot(x, y, ylab="Triceps skinfold thickness in mm (log scale)", xlab="Age in years", axe
axis(3); axis(4); box()
## fit models
fit.poly <- lm(y ~ poly(x)) #polynomial spline
fit.bs <- lm(y ~ bs(x) ) # bspline
fit.ns <- lm(y ~ ns(x) ) #natural spline
fit.sp <- smooth.spline(y ~ x)
## add fit lines to the plot
lines(x, predict(fit.poly, data.frame(x=x)), col=1, lwd=2)
lines(x, predict(fit.bs, data.frame(x=x)), col=2, lwd=2)
lines(x, predict(fit.ns, data.frame(x=x)), col=3, lwd=2)
lines(fit.sp, col=4, lwd=2)
legend(0.05, 3.8, "default values", col=1, bty="n")
par(mar= c(5, 4, vdist, hdist))
plot(x, y, ylab="Triceps skinfold thickness in mm (log)", xlab="Age in years", axes=FALS
axis(1); axis(2); box()
## fit models
fit.poly.4 <- lm(y~ poly(x,4))
fit.bs.4 <- lm(y~ bs(x, df=4) )
fit.ns.4 <- lm(y~ ns(x, df=4) )
fit.sp <- smooth.spline(y~ x, df=4)
## add fit lines to the plot
lines(x, predict(fit.poly.4, data.frame(x=x)), col=1, lwd=2)
lines(x, predict(fit.bs.4, data.frame(x=x)), col=2, lwd=2)
lines(x, predict(fit.ns.4, data.frame(x=x)), col=3, lwd=2)
lines(fit.sp, col=4, lwd=2)
legend(0.05, 3.8, "4 degrees of freedom", col=1, bty="n")
par(mar= c(5, hdist, vdist, 4))
plot(x, y, ylab="Triceps skinfold thickness in mm (log)", xlab="Age in years", axes=FALS
axis(1); axis(4); box()
## fit models
fit.poly.10 <- lm(y~ poly(x,10))
fit.bs.10 <- lm(y~ bs(x, df=10) )
fit.ns.10 <- lm(y~ ns(x, df=10) )
fit.sp <- smooth.spline(y ~ x, df=10)
## add fit lines to the plot
lines(x, predict(fit.poly.10, data.frame(x=x)), col=1, lwd=2)
lines(x, predict(fit.bs.10, data.frame(x=x)), col=2, lwd=2)
lines(x, predict(fit.ns.10, data.frame(x=x)), col=3, lwd=2)
```

```
lines(fit.sp, col=4,lwd=2)
legend(0.05, 3.8, "10 degrees of freedom", col=1, bty="n")
layout(matrix(1, 1, 1))
```

Code 5

```
library(VGAM)
library(gamlss)
set.seed(0)
n <- 400
x <- 0:(n-1) / (n-1)
f <- -3.5+0.2*x^11*(10*(1-x))^6+10*(10*x)^3*(1-x)^10
y <- f + rnorm(n, 0, sd = 2)
fit.gam <- gam(y~ bs(x))
fit.vgam<- vgam(y~ bs(x), uninormal)
fit.gamlss<- gamlss(y~ bs(x), control=gamlss.control(trace=FALSE))

summary(fit.gam)
## Family: gaussian
## Link function: identity
##
## Formula:
## y ~ bs(x)
##
## Parametric coefficients:
##              Estimate Std. Error t value Pr(>|t|)
## (Intercept)  -3.2688     0.4663  -7.010 1.04e-11 ***
## bs(x)1       16.4085     1.3479  12.174  < 2e-16 ***
## bs(x)2       -4.4957     0.8565  -5.249 2.50e-07 ***
## bs(x)3        0.8325     0.7360   1.131    0.259
## ---
## Signif. codes:  0 '***' 0.001 '**' 0.01 '*' 0.05 '.' 0.1 ' ' 1
##
## R-sq.(adj) =  0.498   Deviance explained = 50.2%
## GCV = 5.5952  Scale est. = 5.5393    n = 400
summary(fit.vgam)
## Call:
## vgam(formula = y ~ bs(x), family = uninormal)
##
## Number of linear predictors:    2
##
## Names of linear predictors: mean, loge(sd)
##
## Dispersion Parameter for uninormal family:   1
##
## Log-likelihood: -907.9376 on 795 degrees of freedom
```

```
##
## Number of iterations:  5
##
## DF for Terms and Approximate Chi-squares for Nonparametric Effects
##
##                Df Npar Df Npar Chisq P(Chi)
## (Intercept):1  1
## (Intercept):2  1
## bs(x)          3
summary(fit.gamlss)
## *******************************************************************
## Family:  c("NO", "Normal")
##
## Call:
## gamlss(formula = y ~ bs(x), control = gamlss.control(trace = FALSE))
##
## Fitting method: RS()
##
## -------------------------------------------------------------------
## Mu link function:  identity
## Mu Coefficients:
##              Estimate Std. Error t value Pr(>|t|)
## (Intercept)  -3.2688     0.4640  -7.045 8.30e-12 ***
## bs(x)1       16.4085     1.3411  12.235  < 2e-16 ***
## bs(x)2       -4.4957     0.8522  -5.276 2.19e-07 ***
## bs(x)3        0.8325     0.7323   1.137    0.256
## ---
## Signif. codes:  0 '***' 0.001 '**' 0.01 '*' 0.05 '.' 0.1 ' ' 1
##
## -------------------------------------------------------------------
## Sigma link function:  log
## Sigma Coefficients:
##              Estimate Std. Error t value Pr(>|t|)
## (Intercept)  0.85091    0.03536   24.07   <2e-16 ***
## ---
## Signif. codes:  0 '***' 0.001 '**' 0.01 '*' 0.05 '.' 0.1 ' ' 1
##
## -------------------------------------------------------------------
## No. of observations in the fit:  400
## Degrees of Freedom for the fit:  5
##       Residual Deg. of Freedom:  395
##                       at cycle:  2
##
## Global Deviance:     1815.875
##             AIC:     1825.875
##             SBC:     1845.833
```

```
## *******************************************************************
```

Code 6

```
## Using library(gam)
gam.bs <- gam(y~bs(x))
gam.ns <- gam(y~ns(x,df=3))
gam.bsK <- gam(y~bs(x, knots=seq(0, 1, by=.2)))
gam.nsK <- gam(y~ns(x, knots=seq(0.2, .8, by=.2)))
## define plotting parameters
vdist <- hdist <- 0.2
layout(matrix(1:4, 2, 2, byrow=TRUE), widths= c(10, 10), heights= c(10, 10))
par(mar= c(vdist, 4, 3, hdist))
plot(x, y, ylab="y", xlab="", axes=FALSE)
axis(2); axis(3); box()
lines(x, f, lwd=2, lty=2)
lines(x, predict(gam.bs), col=2, lwd=2)
lines(x, predict(gam.ns), col=3, lwd=2)
legend(0.15,-3,c("Real", "bs", "ns"),
col=c(1, 2, 3), lty=1, bty="n")
par(mar= c(vdist, hdist, 3, 4))
plot(x,y,ylab="",xlab="",axes=FALSE)
axis(3); axis(4); box()
lines(x, f, lwd=2, lty=2)
lines(x, predict(gam.bsK), col=2, lwd=2)
lines(x, predict(gam.nsK), col=3, lwd=2)
legend(0.15,-3,c("Real", "bs", "ns"),
col=c(1, 2, 3), lty=1, bty="n")
legend("topright", "Four interior knots", col=1, lty=0, bty="n")
par(mar= c(5, 4, vdist, hdist))
plot(x, y, ylab="", xlab="x", axes=FALSE)
lines(x, f, lwd=2, lty=2)
axis(1); axis(2); box()
fit.gam <- gam(y~s(x))
t3 <- predict(fit.gam)
lines(x, t3, col=2, lwd=2)
## detach package gam so it does not interfear with mgcv
detach("package:gam", unload=TRUE)
library(mgcv)
fit.mgcv <- gam(y~s(x))
t4 <- predict(fit.mgcv)
lines(x, t4, col=3, lwd=2)
legend(0.12, -2.5, c("Real", "gam/gamlss", "mgcv"), col=c(1, 2, 3), lty=c(2, 1, 1), bty=
legend("topright", "s() function: default values", col=1, lty=0, bty="n")
par(mar= c(5, hdist, vdist, 4))
plot(x, y, ylab="", xlab="", axes=FALSE)
```

```
lines(x, f, lwd=2, lty=2)
axis(1); axis(4); box()
library(gamlss)
gamlss.ps<- gamlss( y~pb(x), control=gamlss.control(trace=FALSE))
detach("package:gamlss")
library(mgcv)
mgcv.ps <-  gam(y~s(x,bs="ps"))
t.gl <- predict(gamlss.ps)
t.g <- predict(mgcv.ps)
lines(x, t.gl,col=2, lwd=2)
lines(x, t.g,col=3, lwd=2)
legend(0.12, -2.5, c("Real", "gamlss", "mgcv"), col=c(1, 2, 3), lty=c(2, 1, 1), bty="n")
layout(matrix(1, 1, 1))
legend("topright", "p-splines: default values", col=1 ,lty=0, bty="n")
```
